# Development and Breeding of Herbicide‐Resistant Sorghum for Effective Cereal‐Legume Intercropping

**DOI:** 10.1002/advs.202503083

**Published:** 2025-05-08

**Authors:** Sanyuan Tang, Jiayang Shi, Xuefeng Li, Mingliang Yang, Chao Li, Dan Zhang, Sen Yang, Cuo Mei, Zuyong Luo, Li Zhang, Wanke Zhang, Chunrui Zhang, Chenbo Zhu, Xiaowei Ma, Ran Xia, Yuhang Chen, Jinsong Zhang, Qingshan Chen, Shouyi Chen, Qi Xie, Feifei Yu

**Affiliations:** ^1^ Institute of Genetics and Developmental Biology The Innovative Academy of Seed Design Chinese Academy of Sciences Beijing 100101 China; ^2^ Cropedit Biotech Co., Ltd. Beijing 102206 China; ^3^ College of Agriculture Northeast Agricultural University Harbin 150000 China; ^4^ University of Chinese Academy of Sciences Beijing 100049 China; ^5^ National Center of Technology Innovation for Maize State Key Laboratory of Crop Germplasm Innovation and Molecular Breeding Syngenta Group China Beijing 102206 China; ^6^ Qilu Zhongke Academy of Modern Microbiology Technology Jinan 250018 China; ^7^ College of Grassland Science and Technology China Agricultural University Beijing 100083 China

**Keywords:** ALS/AHAS, cereal‐legume strip intercropping, herbicide‐resistant sorghum, soybean

## Abstract

Weeds bring a serious challenge to crop production, and herbicides is the most effective and economic way to manage it in field. Sorghum is a critical crop for staple food, fodder, and biofuel. However, the lack of herbicide‐resistant sorghum germplasm severely impedes its production. Here, we conducted a large‐scale screening and identified 13 sorghum mutant lines resistant to imidazolinone (IMI) herbicides. Two unique mutation sites in *SbALS* (*acetolactate synthase*), thus namely *Sbals‐1* (A93T) and *Sbals‐2* (S624N) are discovered, both enhance sorghum tolerance to imazamox. Notably, under high concentrations of imazamox, *sbals‐1* presented a superior growth phenotype and elevated *Sb*ALS activity than *sbals‐2*, a difference that can be attributed to the predicted protein structures. Breeding with *Sbals*, both grain‐ and grass‐type sorghum, shows great weed control and field performance. The herbicide imazamox resistance is further evaluated in a soybean population for sorghum‐soybean strip intercropping, identifying 123 highly resistant soybean varieties. Field intercropping tests indicated health growth of both soybean and sorghum lines post‐imazamox treatment, which enhance field clearance of weed. This study, therefore, provides valuable insights not only for herbicide‐resistant sorghum breeding but also for the successful implementation of efficient and sustainable cereal‐legume intercropping systems.

## Graphical abstract

This study identified two SbALS mutations (A93T and S624N) conferring robust herbicide resistance in sorghum, facilitating efficient weed control. Structural analysis revealed that imazamox resistance is mediated by disrupted herbicide binding. Furthermore, 126 imazamox resistant soybean varieties are screened for sorghum‐soybean intercropping, enhancing weed suppression. These findings provide critical insights for herbicide‐resistant crop breeding and sustainable cereal‐legume intercropping systems.

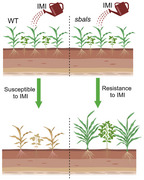

## 1 Introduction

Sorghum (*Sorghum bicolor* [L.] Moench) is a major global cereal crop that provides a stable source of calories for over 500 million people worldwide. It also serves as a crucial source of forage and biofuel. Despite sorghum's superior resistance to multiple abiotic stresses compared with other staple foods like rice, maize, and wheat,^[^ ^1^ ^]^ weeds pose a severe threat to its production. In regions like Arkansas, Kansas, Missouri, Nebraska, South Dakota, and Texas, inadequate weed management leads to average yield losses of 37–61%, translating to annual monetary losses of $7–314 million.^[^ ^2^, ^3^ ^]^ It is therefore crucial to explore effective weed control strategies in sorghum production.

In terms of agriculture management efficiency, the use of low‐cost herbicides is considered the most effective approach for weed control. Imidazolinone (IMI) herbicides, classified as Group B herbicides, are widely utilized in crop production due to their numerous advantages, including broad‐spectrum weed control at low application rates, low soil residual activity, and minimal toxicity to mammals.^[^ ^4^, ^5^, ^6^ ^]^ However, sorghum itself is sensitive to IMI herbicides, and the lack of IMI herbicide‐resistant sorghum varieties currently hampers their use for weed control in sorghum fields.^[^ ^7^, ^8^ ^]^ Therefore, the isolation and breeding of IMI‐resistant sorghum varieties could overcome this limitation and enable effective and cost‐efficient weed control in sorghum fields using IMI herbicides, ultimately benefiting farmers.

Over the past three decades, extensive research has been conducted on the targets of IMI herbicides in plants.^[^ ^9^, ^10^ ^]^ These herbicides primarily target acetolactate synthase (ALS), also known as acetohydroxyacid synthase (AHAS), which is an octameric enzyme consisting of four catalytic and four regulatory subunits.^[^ ^11^ ^]^ ALS plays a crucial role in the biosynthesis pathway of branched‐chain amino acids, including valine, leucine, and isoleucine.^[^ ^12^ ^]^ It catalyzes two parallel reactions: the condensation of two pyruvate molecules to produce acetolactate, leading to the synthesis of valine and leucine, and the condensation of pyruvate and α‐ketobutyrate to yield acetohydroxybutyrate, which is involved in the production of isoleucine.^[^ ^13^, ^14^ ^]^ Inhibition of ALS activity causes a deficiency in these essential amino acids, resulting in decreased protein synthesis and eventual plant death. Understanding the mechanism of action of ALS as the target of IMI herbicides provides valuable insights into their effectiveness and specificity in weed control.

IMI herbicides exert their effects by binding to the substrate access channel of ALS, hindering substrate entry into the active site.^[^ ^15^, ^16^, ^17^ ^]^ Consequently, resistance to IMI herbicides in plants often arises from reduced sensitivity of ALS to IMI binding, while its catalytic activity remains relatively unaffected.^[^ ^15^ ^]^ Point mutations in AHAS/ALS genes have been extensively identified or developed in crops like maize, wheat, rice, oilseed rape, and sunflower through mutagenesis, natural selection, and genome editing strategies.^[^ ^18^, ^19^, ^20^, ^21^ ^]^ Often, a single amino acid mutation is sufficient to confer resistance, and the mutagenesis is genetically dominant. For instance, specific substitutions like A96T, P171L, and A179V (corresponding to *Ta*ALS‐D) in wheat resulted in IMI herbicide resistance.^[^ ^21^, ^22^ ^]^ However, different mutation sites may confer varying degrees of herbicide resistance, and the same mutation may function differently in different species.^[^ ^14^, ^23^ ^]^ Although extensive research has been conducted on the mutation of *ALS* genes in several staple crops such as wheat, rice, and maize, the exploration of *ALS* gene variants conferring herbicide tolerance in sorghum remains relatively limited.

Legume/grass intercropping is a prevalent cropping pattern in southwest China. This practice is widely adopted by farmers due to its ability to maximize the benefits of the symbiotic nitrogen fixation ability of legumes, minimize the excessive consumption of soil water by water‐demanding crops, and improve crop resilience and yield stability.^[^ ^24^, ^25^ ^]^ Additionally, this approach contributes to soil health and biodiversity, enhancing the overall resilience of the agroecosystem.^[^ ^24^ ^]^ Sorghum, with its high tolerance to salinity, drought, and other environmental stresses, along with its high nitrogen‐use efficiency, makes it an ideal candidate for intercropping with soybeans.^[^ ^1^ ^]^ In the context of climate change and increasing demands for sustainable agricultural practices, such intercropping systems present an innovative solution to meet food security while reducing environmental impacts. A key factor contributing to the success of this model is the use of herbicides that are suitable for both intercropping crops. Certain soybean variates have been known to be ALS‐resistant that can be used in this intercropping model.^[^ ^26^ ^]^ Therefore, the development of ALS‐resistant varieties of sorghum becomes a critical piece of the puzzle in enhancing the success of this model, which will extend the benefits of such intercropping systems to areas with different weed pressure profiles, increasing the adaptability and resilience of our agricultural systems.

Here, we isolated IMI‐resistant mutants from mutagenized seed pools with ethyl methanesulfonate (EMS) of sweet sorghum cultivar E048. Comprehensive phenotypic, genetic, and molecular characterizations of the *sbals* mutants unveiled that the substitutions A93T or S624N in *Sb*ALS conferred resistance to IMI herbicides. Our in vitro enzymatic assays demonstrated that the mutated *Sb*ALS exhibited lower sensitivity to IMI inhibition compared to the wild‐type *Sb*ALS. We also screened the high IMI tolerant soybean variants. The practice of cereal‐legume intercropping with one spray of unique herbicide has been shown to bolster crop resilience, enhance ecosystem services, and improve nutrient use efficiency.^[^ ^25^ ^]^ As such, the work presented here, detailing the genetic characterization and underlying mechanism of IMI resistance in sorghum mutants, provides a solid foundation for not only breeding sorghum varieties with herbicide resistance but also advancing the practice of intercropping sorghum with other crops.

## 2 Results

### 2.1 Screening of Imazamox‐Resistant Sorghum Mutants

**Figure 1:**
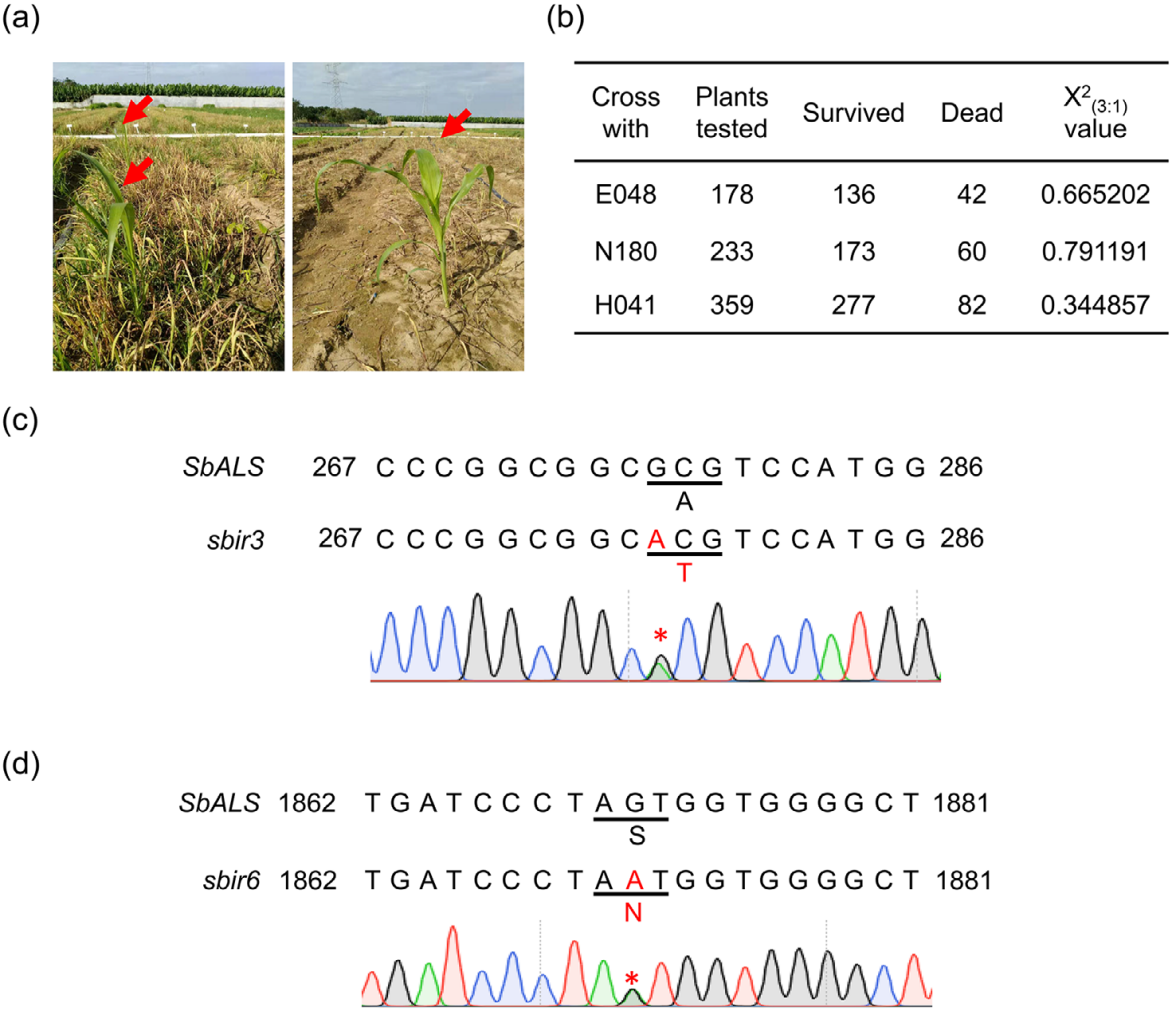
Mutant screening and identification of mutation sites in imazamox‐resistant sorghum lines. a) Field‐based screening of seedlings for mutant identification. The red arrows indicate mutant candidates. b) Segregation pattern of the F_2_ generation derived from backcrossing imazamox‐resistant sorghum lines with E048. c) Amino acid substitution A93T in *Sb*ALS protein resulting from the G277A mutation in imazamox‐resistant sorghum line *sbir3*. *sbir3* exhibits heterozygosity for the G277A mutation in *SbALS*. The numbers indicate the positions of nucleotide bases in the *SbALS* open reading frames. d) Amino acid substitution S624N in *Sb*ALS protein resulting from the G1871A mutation in imazamox‐resistant sorghum line *sbir6*. *sbir6* exhibits heterozygosity for the G1871A mutation in *SbALS*.

A large‐scale screening was conducted to identify imazamox‐resistant sorghum mutants. Sweet sorghum E048 seeds were mutagenized using EMS, resulting in a mutant pool. The M_1_ seeds were planted in the field and self‐bred to obtain M_2_ seeds. From ≈120 000 M_1_ mutant lines, M_2_ seeds were harvested for further analysis. To identify imazamox‐resistant sorghum seedlings, the M_2_ seeds were divided into 1200 pools, with each pool consisting of 100 lines. The sorghum plants were grown in the field and subjected to foliar spraying with a 164.88 g of active ingredient (a.i.) per hectare imazamox solution at the five‐leaf stage, which corresponds to twice the commercially recommended concentration. After a period of 2 to 3 weeks, the majority of the mutagenized sorghum seedlings exhibited sensitivity to imazamox and could not survive. Remarkably, 13 individual plants demonstrated strong survival phenotype after imazamox treatment (**Figure** **1**a), indicating their resistance to the herbicide. These imazamox‐resistant sorghum lines were designated as *sbir1‐13* (*sorghum imazamox‐resistant 1–13*). This successful screening process yielded a diverse set of mutants with enhanced resistance to imazamox, providing a promising foundation for further investigation and utilization of these sorghum lines in weed control.

### 2.2 Identification of the Imazamox‐Resistance Causal Gene in *sbirs*

To determine the mutated gene responsible for imazamox resistance in *sbirs*, M_3_ seeds derived from *sbirs* were collected and subjected to further analysis. The M_3_ seeds were sown in the laboratory and treated with imazamox. It was observed that M_3_ plants from two mutant lines, *sbir3* and *sbir6*, consistently displayed imazamox resistance, indicating their homozygosity for the resistance trait. Genetic analysis of the resistance trait was performed by crossing the M_3_ plants with WT E048 (*Sb*WT), N180, and H041, respectively, resulting in the production of F_1_ plants. The resulting F_1_ plants were then self‐pollinated to generate F_2_ plants. At the five‐leaf stage, the F_2_ plants were treated with 1× imazamox, and the number of herbicide‐resistant and sensitive plants was recorded 10 days after treatment. In total, 178, 233, and 359 plants were analyzed in the three F_2_ populations, of which 136, 173, and 277 were imazamox resistant, while 42, 60, and 82 were imazamox sensitive, respectively (Figure 1b). The observed ratio of resistant to sensitive plants in *sbir3* and *sbir6* was ≈3:1, indicating that the resistance trait is controlled by a single dominant gene.

Given that ALS is responsible for the synthesis of branched‐chain amino acids and serves as the target of imazamox,^[^ ^15^ ^]^ we hypothesized that *ALS* might be mutated in the imazamox‐resistant sorghum lines, *sbirs*. Since the sweet sorghum E048 genome had previously been sequenced and assembled in our lab, the *SbALS* gene sequence was cloned and analyzed using the reference sequence. Protein sequence alignment was also performed between the wild‐type E048 and the imazamox‐resistant alleles (Figure S1, Supporting Information). Notably, we identified two distinct mutant sites that occurred in all 13 imazamox‐resistant lines. Specifically, *sbir3* and five other mutant lines exhibited a G to A mutation at position 277 base pairs (bp) of the coding sequence, resulting in the substitution of the amino acid alanine with threonine (A93T). Similarly, *sbir6* and six other mutant lines displayed a G to A mutation at position 1871, leading to the substitution of the amino acid serine with asparagine (S624N) (Figure 1c,d). Furthermore, it was observed that all imazamox‐resistant individuals in the BC_1_F_2_ generation carried either a homozygous or heterozygous mutation in *SbALS*, while none of the sensitive plants possessed any mutations. These findings confirmed the association between *Sb*ALS point mutations and the imazamox‐resistant phenotype in sorghum. Therefore, the previously screened imazamox‐resistant sorghum mutants, *sbir3* and *sbir6*, were renamed as *sbals‐1* and *sbals‐2*, respectively.

### 2.3 Phenotypic Characterization of *sbals‐1* and *sbals‐2* in Imazamox‐Resisstance

**Figure 2:**
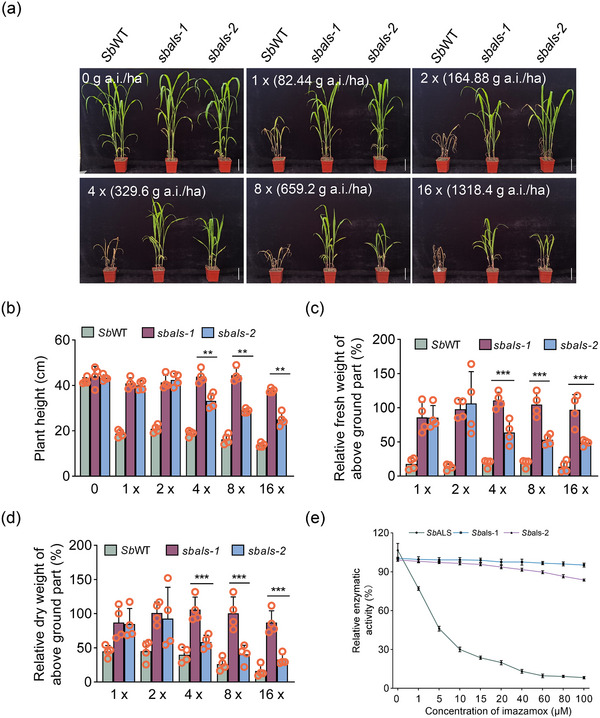
Physiological and biochemical phenotype of *sbals* and *Sb*WT after imazamox treatment. a) Phenotypic observation of *sbals* mutants treated with different concentrations of imazamox. a.i./h, active ingredient (a.i.) per hectare. b–d) Quantification of the plant height, and relative fresh weight and dry weight of aboveground parts in *sbals* mutants. Mean fresh weights and dry weights (% of control) were calculated from three independent biological replicates. Error bars represent the standard deviation (SD). *n* = 4. Asterisks denote statistical differences as determined by Student's *t*‐test: ^**^ *p* < 0.01 and ^***^ *p* < 0.001. e) Effect of imazamox on the activity of wide‐type and mutated versions of *Sb*ALS. The lines depict the fitted line for the mean value of three independent biological replicates corresponding to each data point. Data are presented as the mean ± SD for three biological replicates.

To further assess the imazamox resistance of *sbals‐1* and *sbals‐2*, backcrossing with *Sb*WT was performed, and BC_5_F_3_ homozygous plants were generated (Figure S2a, Supporting Information). Phenotypic experiments were conducted in the greenhouse using different concentrations of imazamox. In the absence of imazamox treatment, both BC_5_F_3_ *Sb*WT/*sbals‐1* and *Sb*WT/*sbals‐2* plants exhibited similar growth to SbWT plants. However, upon imazamox treatment, significant growth inhibition was observed in *Sb*WT plants, even at the recommended field concentration (1×, 82.44 g a.i./ha) (**Figure** **2**a). In contrast, *sbals‐1* and *sbals‐2* plants showed no apparent effect on growth when treated with 1× and 2× imazamox concentrations. Furthermore, they exhibited significantly better growth than *Sb*WT plants in terms of plant height, relative fresh weight, and dry weight after being sprayed with different concentrations of imazamox (Figure 2a–d). Notably, *sbals‐1* and *sbals‐2* plants were able to survive even with 16× imazamox treatment. These results demonstrated that the two BC_5_F_3_ homozygous *Sb*WT*/sbals* mutants exhibited remarkable resistance to imazamox. Additionally, *sbals‐1* with the A93T mutation displayed higher concentration of imazamox resistance compared to *sbals‐2* with the S624N mutation, as evidenced by increased plant height, fresh weight, and dry weight under treatment with 4×, 8×, and 16× imazamox (Figure 2b–d). These findings suggest that the A93T mutation conferred greater imazamox resistance in plants compared to the S624N mutation. Moreover, the enzymatic activities of the mutated *Sb*ALS proteins, including *Sb*als‐1 (A93T), and *Sb*als‐2 (S624N), remained unaffected by varying concentrations of imazamox (Figure 2e). Besides, catalytic efficiency (*k*_cat_/*K*_m_) among *Sb*ALS, *Sb*als‐1, and *Sb*als‐2 has no statistically significant difference (Figure S2b,c, Supporting Information), indicating that the amino acid substitution in the mutants did not alter enzymatic activity of ALS under normal conditions. This insensitivity strongly implies their significant roles in endowing plants with herbicide resistance.

Furthermore, *sbals‐1* and *sbals‐2* were crossed with the elite sweet sorghum N180 to evaluate whether *Sb*als‐1 (A93T) and *Sb*als‐2 (S624N) could contribute to imazamox resistance in the N180 background. After five generations of backcrossing, BC_5_F_1_ plants were self‐pollinated to generate BC_5_F_3_ *Sb*als‐1 (A93T) and *Sb*als‐2 (S624N) homozygous plants. Our results showed that the BC_5_F_3_ *sbals‐1* and *sbals‐2* plants in the N180 background also displayed imazamox resistance compared to N180 (Figure S2d–g, Supporting Information). Additionally, N180*^als‐1^* exhibited a superior growth phenotype compared to N180*^als‐2^* (Figure S2d–g, Supporting Information), which was consistent with the result obtained from backcrossing with E048.

In addition, to determine the herbicide resistance spectrum of *sbals‐1* and *sbals‐2*, we conducted an assay using various of *ALS*‐inhibiting herbicides at different concentrations. The herbicides used included the IMI herbicides imazapic, the triazolopyrimidine sulfonamide (TP) herbicide pyroxsulam, the sulfonylaminocarbonyltriazolinone (SCT) herbicide flucarbazone‐sodium, the pyrimidinylthiobenzoate (PTB) herbicide bispyribac‐sodium and the sulfonylurea (SU) herbicides nicosulfuron. Each herbicide was sprayed at 1×, 2×, and 4× the commercially recommended field application dose. Compared to the wide‐type, both *sbals‐1* and *sbals‐2* showed resistance to imazapic, pyroxsulam, and flucarbazone‐sodium, but not to bispyribac‐sodium and nicosulfuron (Figure S3, Supporting Information). These results suggest that *sbals‐1* and *sbals‐2* have the potential to confer broad‐spectrum herbicide resistance.

### 2.4 Structural Basis of Imazamox Resistance Difference Between *Sb*als‐1 and *Sb*als‐2

To elucidate the molecular basis of imazamox resistance in *sbals‐1* and *sbals‐2*, we employed AlphaFold‐based structural modeling to predict the sorghum ALS structure in complex with imazamox. The predicted dimeric ALS structure exhibited high similarity to Arabidopsis ALS (PDB ID: 1z8n) complexed with imazaquin,^[^ ^16^ ^]^ with a root mean square deviation (RMSD) of 0.6 Å for 1140 superimposed Cα atoms. Further AlphaFold modeling of the A93T and S624N mutants of *Sb*ALS revealed that the structures were nearly indistinguishable from the wild type, suggesting that these herbicide‐resistant mutations did not induce significant conformational changes in the protein. Considering that imazamox and imazaquin both belong to the imidazolinone herbicide class and share similar imidazole moieties in their molecular structures, we anticipated that they would occupy comparable binding sites in homologous ALS proteins.

**Figure 3:**
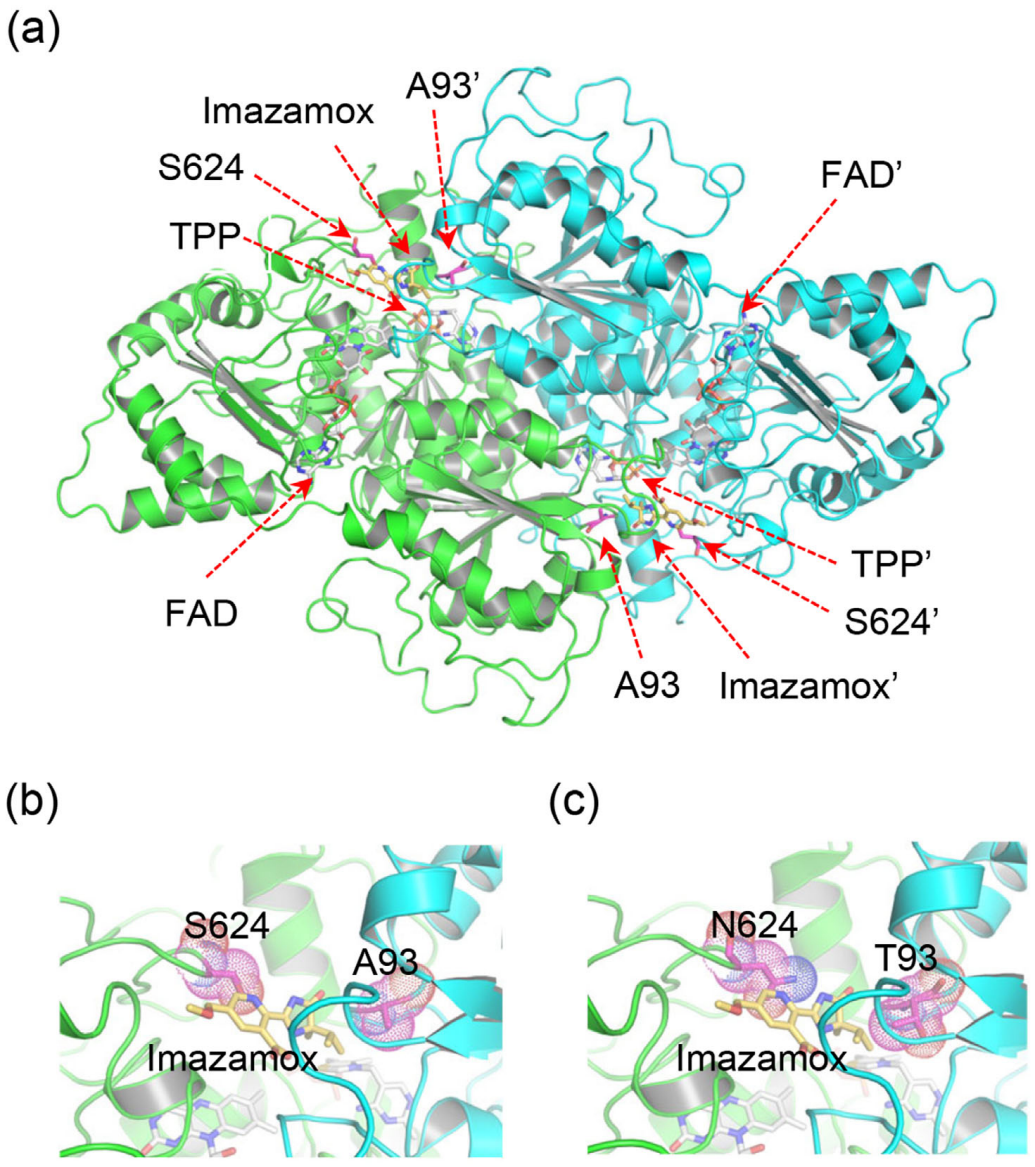
AlphaFold‐based modeling of *Sb*ALS in complex with imazamox. a) Ribbon representation of the modeled *Sb*ALS‐imazamox complex. The protomers in the dimeric structure are depicted in green and cyan, respectively. Two residues (A93 and S624) identified in the herbicide‐resistant screen, the cofactors FAD, TPP, and the herbicide imazamox are shown by sticks. b,c) Zoomed‐in views of the imazamox binding site, wild type structure (b) and two herbicide‐resistant mutant sites (c). Two residues (A93 and S624) identified in the herbicide‐resistant screen shown by purplish red sticks.

Next, we performed molecular docking of imazamox into the herbicide binding site based on the structure of the Arabidopsis ALS‐imazaquin complex (**Figure** **3**a). The imidazolinone herbicide binds to a pocket formed by 12 residues at the dimeric interface, and these interactions are vital for anchoring the herbicide to the ALS protein.^[^ ^16^ ^]^ In sorghum ALS, residue A93 establishes hydrophobic contacts with the isopropyl and methyl groups of the dihydroimidazolone ring in imazamox (Figure 3b). However, in the A93T mutant, the enlarged side‐chain of threonine substitution obstructs the binding site, resulting in the displacement of the herbicide (Figure 3c). Similarly, residue S624 (located on the other protomer) interacts with the opposite side of the imazamox molecule within the herbicide binding pocket (Figure 3b). In the S624N mutant, the substitution of serine to asparagine creates a barrier that hinders the aromatic ring of the imazamox molecule from binding (Figure 3c). Overall, the A93T and S624N mutations in *Sb*ALS do not induce conformational changes in the protein but rather generate impediments within the herbicide binding pocket, preventing imazamox binding and conferring herbicide resistance in sorghum.

### 2.5 Field test of Improved Sorghum Varieties Bred with the *sbals*

**Figure 4:**
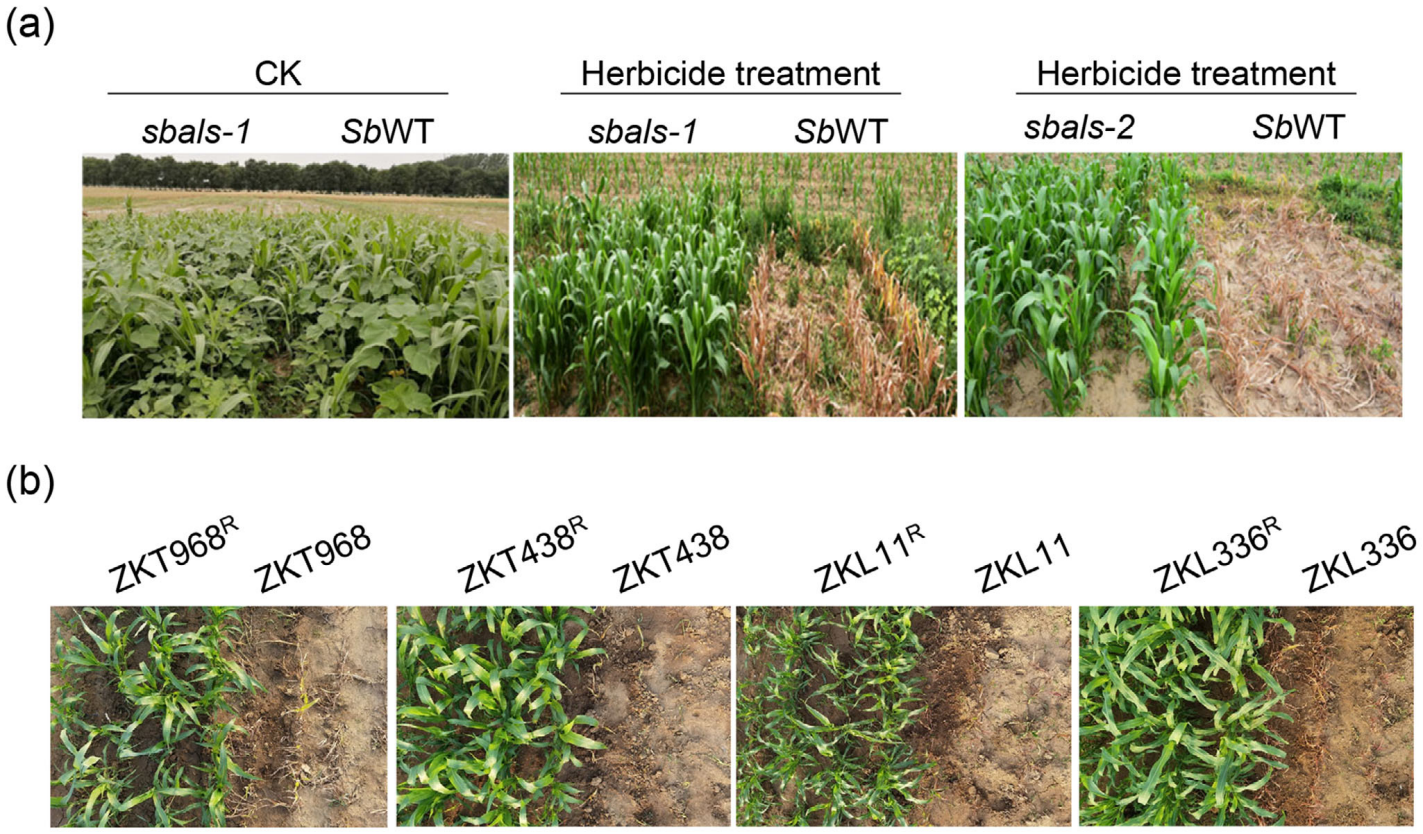
Herbicide responses of *sbals* mutants and their intercropping with identified herbicide‐resistant soybean varieties in the field. a) Phenotype of the identified *sbals* mutant lines with E048 (*Sb*WT) background with and without imazamox spraying in Beijing. b) Herbicide responses of the improved hybrid varieties ZKT968^R^, ZKT438^R^, ZKL11^R^, and ZKL336^R^. The seedlings were sprayed with a 2 × concentration of imazamox and photographed after 10 days.

The field tests were conducted to evaluate the performance of new sorghum varieties bred using the *sbals‐1* and *sbals‐2* mutants. Remarkably, the *sbals‐1* and *sbals‐2* mutants displayed high resistance to herbicides at high concentrations, and no adverse effects on plant growth and production were observed, with or without imazamox spraying during the field test in Beijing and Hainan (**Figure** **4**a; Figure S4, Supporting Information).

Inspired by these promising results, we leveraged the valuable *sbals‐1* mutants as genetic resources for developing herbicide‐resistant sorghum varieties. To assess the stability of the A93T mutation in different sorghum backgrounds and to create additional hybrid sorghum varieties with herbicide resistance, we performed backcrossing to introduce the A93T locus from *sbals‐1* into four hybrid sorghum varieties (ZKT968, ZKT438, ZKT060, and ZKL11). The *sbals‐1* was used as the donor parent, and we crossed it with the maintainer and restorer lines of the respective hybrid varieties as recurrent parents. The resulting progenies were backcrossed up to the BC_5_F_1_ generation. From the BC_5_F_1_ population, we selected self‐pollinated plants to obtain BC_5_F_2_ seeds. By conducting herbicide‐spraying and confirming the presence of the A93T mutation through molecular verification, we identified homozygous lines with stable physiological traits. To confer herbicide resistance to the hybrid varieties, we crossed sterile lines of each hybrid variety with corresponding homozygous maintainer lines to generate the BC_1_F_1_ generation. Subsequently, the recurrent parents (maintainer lines) were utilized for further backcrossing. In each generation, we selected resistant plants with vegetative growth and appearance like the recurrent parent for the next round of backcrossing, continuing until the BC_2_F_1_ generation. Again, we identified homozygous lines with stable physiological traits by conducting herbicide‐spraying and then confirmed by molecular verification of the A93T locus. Ultimately, homozygous sterile lines of the hybrid varieties were crossed with corresponding homozygous restorer lines to obtain herbicide‐resistant hybrid varieties, namely ZKT968^R^, ZKT438^R^, ZKL11^R^, and ZKL336^R^.

Subsequently, the improved hybrid varieties ZKT968^R^, ZKT438^R^, ZKL11^R^, and ZKL336^R^ were field‐planted and subjected to imazamox spraying at the five‐leaf stage to evaluate their imazamox‐resistant phenotype. In contrast to the imazamox‐sensitive parent lines (ZKT968, ZKT438, ZKL336, and ZKL226), all the varieties carrying *Sb*als‐1 (A93T) exhibited survival under herbicide spraying and displayed normal growth (Figure 4b; Figure S5, Supporting Information). These findings demonstrate the consistent stability and effectiveness of the *Sb*als‐1 (A93T) mutation across different sorghum backgrounds, underscoring its potential for breeding imazamox‐resistant varieties.

### 2.6 Screening of Imazamox‐Resistant Soybean Population

**Figure 5:**
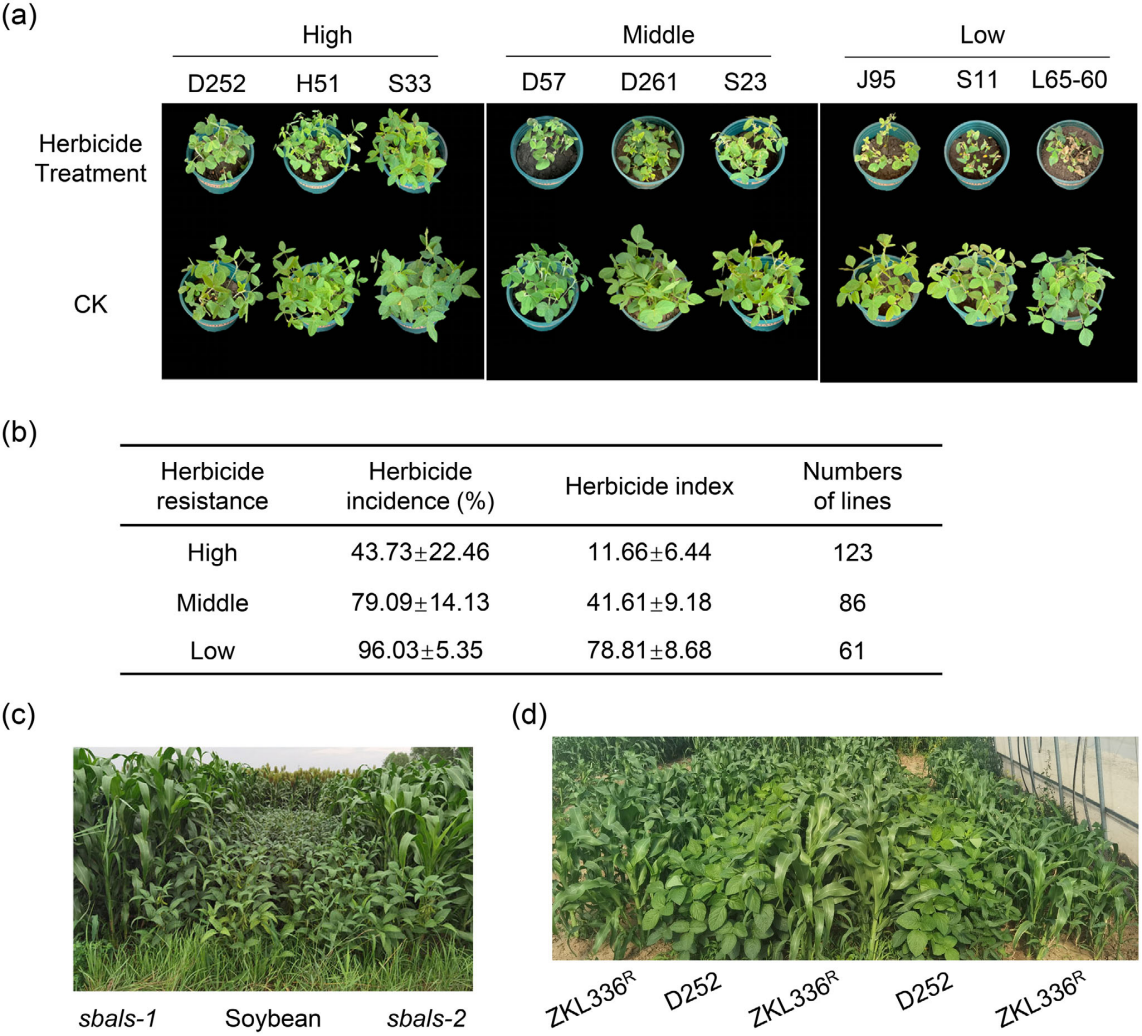
Screening of imazamox‐resistant soybean lines and their intercropping with identified herbicide‐resistant *sbals* sorghum lines in the field. a) Representative soybean accessions with different levels of imazamox resistance. A soybean population containing 270 accessions was treated with imazamox to screen for resistance lines. All the plants were photographed 10 days after imazamox spraying. The level of resistance was categorized as high tolerance, medium tolerance, and low tolerance based on the herbicide resistance index. D252, Donnong252. H51, Heilongjiang51. S33, Suinong33. D57, Dongnong57. D261, Dongnong261. S23, Suinong23. J95, Jiyu95. S11, Suinong11. CK, control group. b) Analysis of variance (ANOVA) on the damage of rate and index of different resistant soybean germplasm. c) Intercropping of herbicide‐resistant soybean lines with *sbal*s‐1 and *sbals‐2* in field. All plants were photographed 10 days after imazamox spraying. d) Intercropping of herbicide‐resistant soybean D252 with sorghum plants derived from the cross between *sbals‐1* and elite sorghum varieties in greenhouse. Homozygous offspring of the BC_6_F_3_ generation of ZKL336^R^ were intercropped with soybean, and all plants were photographed 10 days after imazamox spraying.

Cereal‐legume intercropping has been recognized as an effective strategy for sustainable agriculture, as it enhances crop yield and nutrient utilization.^[^ ^27^, ^28^ ^]^ However, the lack of common herbicides suitable for both cereals and legumes pose a significant challenge to cereal‐legume intercropping worldwide. In this context, the breeding of herbicide‐resistant sorghum opens new possibilities for cereal‐legume intercropping for forage propose. To facilitate intercropping between sorghum and soybean, we then screened soybean lines for imazamox tolerance in a population of 270 lines under greenhouse conditions. Various levels of imazamox tolerance were identified, including high, moderate, and low tolerance (**Figure** **5**a; Figure S6, Supporting Information). The results of resistance assessment showed varying degrees of herbicide damage among different soybean germplasm resources. Among the 270 tested soybean germplasms, 123 exhibited high resistance to imazamox, accounting for 45.56% of all germplasm resources. There were 86 germplasms with moderate resistance, accounting for 31.85%, while 61 germplasms were relatively sensitive to imazamox, representing 22.59% of the total (Figure 5b).

Highly resistant germplasms, such as Dongnong 252, Heinong 51, Suinong 33, Aika 166, and Heinong 37, exhibited herbicide indices ranging from 0 to 10 and herbicide incidences between 0% and 45% (Table S1, Supporting Information). Treated soybean plants had minimal differences in plant morphology and only slight inhibition of leaf area compared to the control group (Figure 5a; Figure S6a, Supporting Information). Moderately resistant germplasms, including Dongnong 57, Dongnongdou 261, Suinong 23, LS201, and Vinton, displayed herbicide indices ranging from 30 to 60, with herbicide incidences ≈79.09 ± 14.13%. Imazamox initially suppressed the development of stem tips in these germplasms, but the inhibition was relieved after 4–7 days, leading to the emergence of new trifoliate leaves at the stem tips (Figure 5a; Figure S6b, Supporting Information). Highly sensitive germplasms, such as Jiyu 95, Suinong 11, L65‐60, L63‐1397, and Hefeng 25, showed herbicide indices above 90 and herbicide incidences reaching 100%. The herbicide damage in these germplasms was severe, with significant differences in plant morphology compared to the control group (Figure 5a; Figure S6c, Supporting Information). The growth of stem tips was arrested, resulting in wilting, and yellowing of the entire plant, and in severe cases, the plants withered and died.

### 2.7 Contribution of Imazamox‐Resistant Sorghum Breeding to Cereal‐Legume Intercropping

Based on the analysis of imazamox tolerance in different soybean lines and the breeding of imazamox‐resistant sorghum varieties, we selected a highly imazamox‐resistant soybean variety (Dongnong 252) for intercropping with *sbals‐1* and *sbals‐2* lines. The field test results demonstrated successful growth of both sorghum and soybean after herbicide spraying (Figure 5c). Furthermore, the herbicide‐resistant sorghum elite varieties ZKL336*^als‐1^* were selected for intercropping with the highly imazamox‐tolerant soybean variety Dongnong 252. The planting scheme involved two rows of sorghum followed by two rows of soybeans, with imazamox spraying at the five‐leaf stage of sorghum growth (Figure 5d). Importantly, all the sorghum and soybean plants survived the herbicide spraying and exhibited normal growth. These findings highlight the significance of developing and promoting herbicide‐resistant sorghum varieties, and the successful intercropping strategy between herbicide‐resistant sorghum and soybean holds great promise for the development of sustainable intensive agriculture.

## 3 Discussion

The presence of weeds poses a significant threat to crop yields due to their ability to outcompete crops for resources and inhibit their growth.^[^ ^2^, ^29^, ^30^, ^31^ ^]^ Effective weed control is crucial for maintaining crop productivity. Especially for sorghum, slow growth at seedling stage and no selective herbicide to control weeds at early stage usually cost a lot of labor in sorghum production. Herbicide‐based weed management is considered more efficient in terms of energy and cost compared to physical or biological methods. However, the development of herbicide‐resistant sorghum varieties has been limited, highlighting the need for further research in this area. In this study, we successfully identified and analyzed herbicide‐resistant sorghum alleles, providing valuable insights for the development of herbicide‐resistant sorghum varieties. Moreover, the breeding of herbicide‐resistant sorghum opens possibilities for sorghum‐soybean intercropping, as demonstrated in our field tests. This intercropping system has the potential to enhance weed control and optimize crop productivity, contributing to sustainable agricultural practices.

In our study, we discovered that two distinct mutations in the *SbALS* gene conferred high resistance to IMI herbicides in sorghum plants, resulting in a resistance level of at least 16‐fold compared to susceptible plants. This is the first identification of a single mutation in the *SbALS* gene that confers IMI herbicide resistance in sorghum, distinguishing from the mutations in patent document applied by DuPont® for herbicide‐tolerant sorghum discovered from a wild sorghum, which carries a double mutation in the *SbALS* gene (Patent No. WO2008/073800 A3, https://webofscience.clarivate.cn/wos/alldb/full‐record/DIIDW:2008H31853). Notably, while both *sbals‐1* and *sbals‐2* mutants exhibit high IMI resistance, *sbals‐1* appears to be more robust in terms of plant height and biomass after exposure to high concentrations of IMI herbicides. This observation aligns with previous studies showing that specific ALS resistance mutations can vary in their strength.^[^ ^22^, ^32^ ^]^ It should be noted that neither mutation compromises the enzyme's catalytic efficiency under herbicide‐free conditions. The enhanced herbicide resistance observed in the mutants is thus likely attributable to altered herbicide binding rather than impaired enzymatic function. The phenomenon of differential herbicide resistance conferred by diverse ALS mutation sites have also been reported in other crops. For instance, in wheat, m‐*Ta*ALS‐D (A96T) exhibits higher resistance to imazethapyr compared with the m‐*Ta*ALS‐D (S627N) mutation. In rice, a novel ALS mutation site, W548M, exhibits greater resistance to multiple herbicide classes (such as IMI, SU, SCT) than S627N mutation.^[^ ^22^, ^23^ ^]^ These studies illustrate that distinct ALS mutation sites endow crops with different levels of herbicide resistance, highlighting the significance of screening for mutation sites with stronger resistance in herbicide‐tolerance research. The potential synergistic effects of integrating the A93T and S624N mutations and their ability to confer resistance to other herbicides, as observed in rice and wheat, warrant further investigation.^[^ ^22^, ^23^ ^]^ Furthermore, the herbicide resistance in our sorghum *sbals* mutants exhibited a dominant trait, and the heterozygous mutant lines displayed strong IMI resistance while maintaining normal growth, making them suitable for production tri‐hybrid seeds and offering potential benefits in terms of reduced time and labor costs in the breeding process.

In recent years, the development of transgenic technology and genome editing has enabled the creation of various herbicide‐resistant crops.^[^ ^20^, ^21^, ^33^, ^34^, ^35^ ^]^ While these advanced techniques offer the potential for generating transgene‐free crops, their application is often constrained by the genetic background of the recipient plants and so as the regulatory policies of different countries. In contrast, the use of chemically mutagenized mutant pools for screening herbicide‐resistant crops presents certain advantages over gene editing techniques and is therefore frequently employed.^[^ ^36^, ^37^, ^38^ ^]^ Chemically mutagenized lines avoid transgenic safety concerns that restrict large‐scale planting of genetically modified crops. Moreover, it can quickly develop herbicide‐resistant varieties based on existing elite alleles, regardless of genetic background, and without the need for complex genetic engineering.

The findings of this study have significant implications for cereal‐legume intercropping in agriculture and practical crop production. By incorporating the screened IMI‐resistant soybeans with the herbicide‐resistant sorghum mutants, field tests demonstrated successful weed control in sorghum‐soybean intercropping through the application of herbicides. This breakthrough overcomes the previous limitation of incompatible herbicides and provides a promising solution for farmers practicing cereal‐legume intercropping. Moreover, the nutritional benefits of the sorghum‐soybean mixed silage further enhance the significance of this study. The mixed silage derived from the intercropped sorghum and soybean contains higher levels of starch, protein, and fiber compared to sole sorghum silage or soybean silage, improving the palatability and nutritional value of livestock feed.^[^ ^26^, ^39^ ^]^ In addition to practical applications, this study provides vital information for the commercial breeding of IMI‐resistant sorghum varieties. The identification and characterization of the herbicide‐resistant *sbals‐1* and *sbals‐2* mutants serve as valuable genetic resources for future breeding efforts in developing sorghum varieties with enhanced weed management capabilities.

Overall, this research significantly advances cereal‐legume intercropping strategies by addressing the challenge of weed control through the development of herbicide‐resistant sorghum mutants. It offers practical solutions to improve yields, minimize risks, and enhance the nutritional value of livestock feed. Furthermore, it highlights the importance of considering herbicide compatibility in intercropping systems, paving the way for further optimization and exploration of cereal‐legume intercropping strategies in agricultural practices.

## 4 Experimental Section

### Isolation of IMI‐Resistant Lines

M_2_ seeds of total 1000 pools with 100 line per pool were applied to screen for IMI‐resistant mutants, ≈500 g of seeds from each pool of the M_2_ mutant library were directly sown in separate plots. When the plants reached the 3–4 leaf stage, they were uniformly sprayed with a solution of imazamox (2×, 164.88 g a.i./ha). Three weeks after herbicide spraying, the plant phenotype was evaluated, and plants with a green, healthy heart leaf were identified as resistant plants.

### Genomic DNA Extraction, PCR Amplification, and Sequence Analysis

DNA extraction was performed using the CTAB method from 2‐week‐old plants. PCR reactions were set up with 30 µL reaction mixtures, consisting of 100 ng of template DNA, 0.4 µm of primers, and 1 U of 2× Flash master mix (Cwbio, Jiangsu, China). Gel extraction of DNA was carried out using the Wizard® SV Gel and PCR Clean‐Up System (Promega, Madison, USA). The obtained DNA or protein sequences were aligned following sequencing. The primers used for PCR amplification are listed in Table S2 (Supporting Information).

### Breeding of Improved Sorghum Varieties with Herbicide‐Resistant Mutant *sbals‐1*

To ensure the genetic integrity of herbicide‐resistant mutant *sbals‐1*, backcrossing was conducted with the wild‐type varieties E048 and N180, respectively, to eliminate any other EMS‐induced mutations that might affect the herbicide‐resistant mutant's growth and development. In the backcrossing process, the herbicide‐resistant mutant *sbals‐1* was used as the male parents and crossed them with the wild‐type E048 and N180 as the recurrent female parents. The backcrossing was diligently carried out until the BC_5_F_1_ generation. At each generation of backcrossing, herbicide spraying with 1.2 mm imazamox was performed to selectively eliminate the non‐resistant plants. From the BC_5_F_1_ population, plants were self‐pollinated to obtain BC_5_F_2_ seeds. Among the BC_5_F_2_ individuals, that exhibited the closest resemblance in appearance to the recurrent parent were carefully selected for PCR analysis to confirm the presence of the desired mutation. Plants with homozygous mutations were further self‐pollinated to propagate the BC_5_F_3_ seeds. The BC_5_F_3_ plants were subjected to meticulous evaluation to assess their level of herbicide resistance and the stability of other important agronomic traits. This evaluation allowed us to ensure that the herbicide‐resistant traits were effectively transferred and stabilized in the backcrossed populations. By following these rigorous breeding and selection procedures, BC_5_F_3_ plants with the desired herbicide‐resistant mutations were obtained while maintaining the essential agronomic traits required for the growth and development of the herbicide‐resistant sorghum varieties.

### Characterization of Herbicide Tolerance

In the greenhouse, seeds from N180, N180*^als‐1^*, and N180*^als‐2^*, were sown in 8 cm pots in the greenhouse. When the plants reached the four‐leaf stage, they were uniformly sprayed with imazamox at concentrations of 82.44, 164.88, 329.6, 659.2, and 1318.4 g a.i./ha, the the IMI herbicides imazathapyr, the TP herbicide pyroxsulam, the SCT herbicide flucarbazone‐sodium, the PTB herbicide bispyribac‐sodium and the SU herbicides nicosulfuron at 1×, 2×, and 4× recommended field dosage. After 10–14 days of treatment, a phenotypic analysis was conducted. Plant height was measured for each line at different concentrations using three independent biological replicates. The reported mean fresh weights and dry weights were also based on three independent biological replicates. For the field test of improved sorghum varieties, at the five‐leaf stage, the plants were treated with 2× imazamox, and the phenotype was recorded 10 days after treatment.

### Expression and Purification of *Sb*ALS‐His Fusion Proteins Using *Escherichia coli*

To express and purify the His‐*Sb*ALS fusion proteins, *E. coli* strain BL21 (DE3) was used. RNA was extracted from E048 or the *sbals* mutants, and cDNA was obtained by reverse transcription. The cDNA was used as a template to amplify the different mutant forms of *SbALS* sequences. The PCR products were then fused with pET‐28a (+) plasmids digested with *Bam*H I and *Hind* III. The resulting plasmids were transformed into *E. coli* and used to inoculate 5 mL of LB medium for overnight shaking to generate a 200 mL culture. Once the OD_600_ of the *E. coli* culture reached 0.6–0.8, the expression of the target protein was induced by adding 0.1 mm IPTG and incubating for 18 h at 16 °C. The cells were harvested by centrifugation at 5000 g, resuspended in lysis buffer (300 mm NaCl, 50 mm NaH_2_PO_4_, 10 mm imidazole, pH 7.0), and lysed using ultra‐high pressure. Cell debris was removed by centrifugation at 20 000 g. The protein was purified using Ni‐NTA agarose (Qiagen, Hilden, Germany). Non‐specifically bound proteins were washed away using lysis buffer containing 20 mm imidazole. Elution buffer (10 mm NaH_2_PO_4_, 150 mm NaCl, 300 mm imidazole, pH 7.0) was used to elute the target fusion protein. The protein samples were supplemented with 50% (v/v) glycerol and stored at −80 °C until further enzymatic assays. The protein concentration was determined using the Bradford Reagent (Bio‐Rad Laboratories, Inc., Hercules, CA, USA) with a standard curve based on bovine serum albumin. The primers used for PCR are listed in Table S2 (Supporting Information).

### In Vitro Assay for ALS Activity

To assess ALS activity, a 37.5‐µL reaction mixture was prepared, consisting of 10 mm MgCl_2_, 10 mm thiamine pyrophosphate, 100 mm potassium phosphate buffer (pH 7.0), 1% (v/v) DMSO, 100 mm sodium pyruvate, 0.1 mm flavin adenine dinucleotide disodium salt hydrate, and 3 µL elution buffer containing 3.75 µg protein. The ALS inhibitor used in this study was imazamox (Macklin, Shanghai, China).

Different concentrations of inhibitors dissolved in DMSO were added to the reaction mixture. After incubating the mixture at 37 °C for 1 h, the reaction was stopped by adding 7.5 µL of 5% H_2_SO_4_. Subsequently, the acetolactate produced was decarboxylated to acetoin by incubating for 15 min at 60 °C. To develop color, 31.25 µL of α‐napthol (50 g L^−1^ in 4 M NaOH) and 31.25 µL of creatine (5 mg mL^−1^) were added to the reaction mixture and incubated for another 15 min at 60 °C. The absorbance of the samples was measured at 540 nm using a microtiter plate reader (Varioskan Flash; Thermo Fisher, Waltham, MA, USA). Data from each line were analyzed using a nonlinear regression model in Sigmaplot 14.0.

### 3D Structure Modeling Using AlphaFold

Briefly, protein sequences of *Sb*ALS were run with the local‐installed AlphaFold2 program^[^ ^40^ ^]^ and the CASP14 preset and databases (as of Nov 24, 2021). The top five generated dimer models had model confidence (0.8*ipTM+0.2*pTM) scores ranging from 0.907 to 0.895. All five AlphaFold‐predicted *Sb*ALS dimer structures were almost identical, with their root means square deviations (RMSD) less than 0.50 Å for ≈1180 superimposed Cα. The top‐rank dimer model (scored 0.907) was used for further construction of flavin‐adenine dinucleotide (FAD), thiamine diphosphate (TPP), and herbicide (imazamox) into the *Sb*ALS, based on the structure of the Arabidopsis ALS‐imazaquin complex (PDB ID: 1z8n, sequence identity 75%). The AlphaFold‐predicted structures of the imazamox‐resistant mutants share nearly identical structures to that of wild‐type, with their root mean square deviations (RMSD) of 0.34 Å/1184 superimposed Cα, and 0.69 Å/1190 superimposed Cα for A93T and S624N, respectively.

### Soybean Growth Conditions and Herbicide Treatment

The study employed imazamox as the herbicide of interest and evaluated its effectiveness on 270 different soybean germplasm resources in the field. The experimental trials were conducted at the Xiangyang Experimental Station of Northeast Agricultural University (126°18′E, 45°36′N) on June 6, 2021. The experimental design followed a completely randomized block design with three replications. Each row measured 0.5 m in length and 0.66 m in width, with six holes per row and two seeds per hole. Field management practices adhered to standard agricultural protocols. The herbicide solution was prepared as per the manufacturer's instructions, with a concentration of 70 mg of imazamox per 20 L of water. The solution was uniformly sprayed on soybean plants at the fully expanded leaf stage, which occurred on June 17. To prevent herbicide drift and minimize any potential impact on the experimental results, the control group was covered with plastic sheets during the spraying process.

### Evaluation of Imazamox Phytotoxicity in Soybean Varieties

Phytotoxicity assessment was performed on the 8th day following the application of the imazamox. The phytotoxicity level of each plant was evaluated according to the phytotoxicity grading standard for pesticides in the agricultural industry of the People's Republic of China. The classification criteria for phytotoxicity levels are as follows: Level 0 corresponds to sprayed plants that exhibit growth similar to the control group. Level 1 indicates slight differences in plant height and leaf color compared to the control group. Level 2 was assigned to treated plants that showed slight deformities and were shorter in height compared to the control group. Level 3 was characterized by noticeable stunting, thicker stems, slightly thicker and darker leaves, or yellowing of leaves. Level 4 refers to plants that had ceased growth, exhibit severe deformities, withered or completely yellowed leaves, and wilting. Finally, Level 5 was assigned to plants that had died (Table S1, Supporting Information).

The phytotoxicity incidence and phytotoxicity index were calculated to quantify the extent of imazamox‐induced damage. The phytotoxicity incidence represents the percentage of plants exhibiting herbicide phytotoxicity among the total number of surveyed plants, calculated as: Phytotoxicity incidence = (number of plants with phytotoxicity / total number of surveyed plants) × 100%. The phytotoxicity index reflects the degree of soybean damage caused by the herbicide, calculated as: Phytotoxicity index = ∑ (individual level × number of plants) / (total number of examined plants × highest level) × 100. In this study, soybean resistance was assessed based on the phytotoxicity index, and soybean plants were classified into three levels of resistance: resistant (0 ≤ phytotoxicity index ≤ 30), moderately resistant (30 < phytotoxicity index ≤ 60), and sensitive (60 < phytotoxicity index ≤ 100).

### Intercropping Assay of Sorghum and Soybean

An intercropping assay was conducted using IMI‐resistant sorghum varieties in plots measuring 5 m in length with a spacing of 4 m between each plot. The intercropping arrangement consisted of six rows of sorghum and four rows of soybean. Two different soybean varieties were utilized, with each variety planted in two rows. The inter‐row distance between sorghum and soybean was set at 30 cm, and the distance between adjacent sorghum and soybean rows was also 30 cm. Both sorghum and soybean were sown simultaneously. At the five‐leaf stage of sorghum growth, an imazamox solution was sprayed at a concentration of 82.44 g a.i./ha. Phenotypic observations and photographs were taken after a 2‐week period following the treatment.

## Conflict of Interest

The authors declare no conflict of interest.

## Author Contributions

S.T. and J.S. contributed equally to this work as co‐first authors of this article. Q.X., S.T., and F.Y. conceived and designed the research. S.T., S.J., X.L., M.Y., C.L., S.Y., C.M., Z.L., L.Z., C.Z., and C.Z. performed the experiments. F.Y., Q.X., S.J., S.T., W.Z., R.X., Y.C., J.Z., Q.C., and S.C. analyzed the data. F.Y. and S.J. wrote the paper. F.Y., Q.X., and X.M. revised the paper. All authors approved the final manuscript.

## Supporting information



Supporting Information

Supplemental Tables

## Data Availability

Research data are not shared.
