# Supplementary material for: Development and Breeding of Herbicide‐Resistant Sorghum for Effective Cereal‐Legume Intercropping
Source: Adv Sci (Weinh). 2025 May 8;12(27):2503083. doi: 10.1002/advs.202503083 (PMC12279207; doi:10.1002/advs.202503083)
Supplement: Supplementary file 1 — Supporting Information [file ADVS-12-2503083-s001.pdf]

ADVANCED SCIENCE
Open Access

Supporting Information

for *Adv. Sci.*, DOI 10.1002/advs.202503083

Development and Breeding of Herbicide-Resistant Sorghum for Effective Cereal-Legume Intercropping

Sanyuan Tang, Jiayang Shi, Xuefeng Li, Mingliang Yang, Chao Li, Dan Zhang, Sen Yang, Cuo Mei, Zuyong Luo, Li Zhang, Wanke Zhang, Chunrui Zhang, Chenbo Zhu, Xiaowei Ma, Ran Xia, Yuhang Chen, Jinsong Zhang, Qingshan Chen, Shouyi Chen, Qi Xie and Feifei Yu**

Figure S1

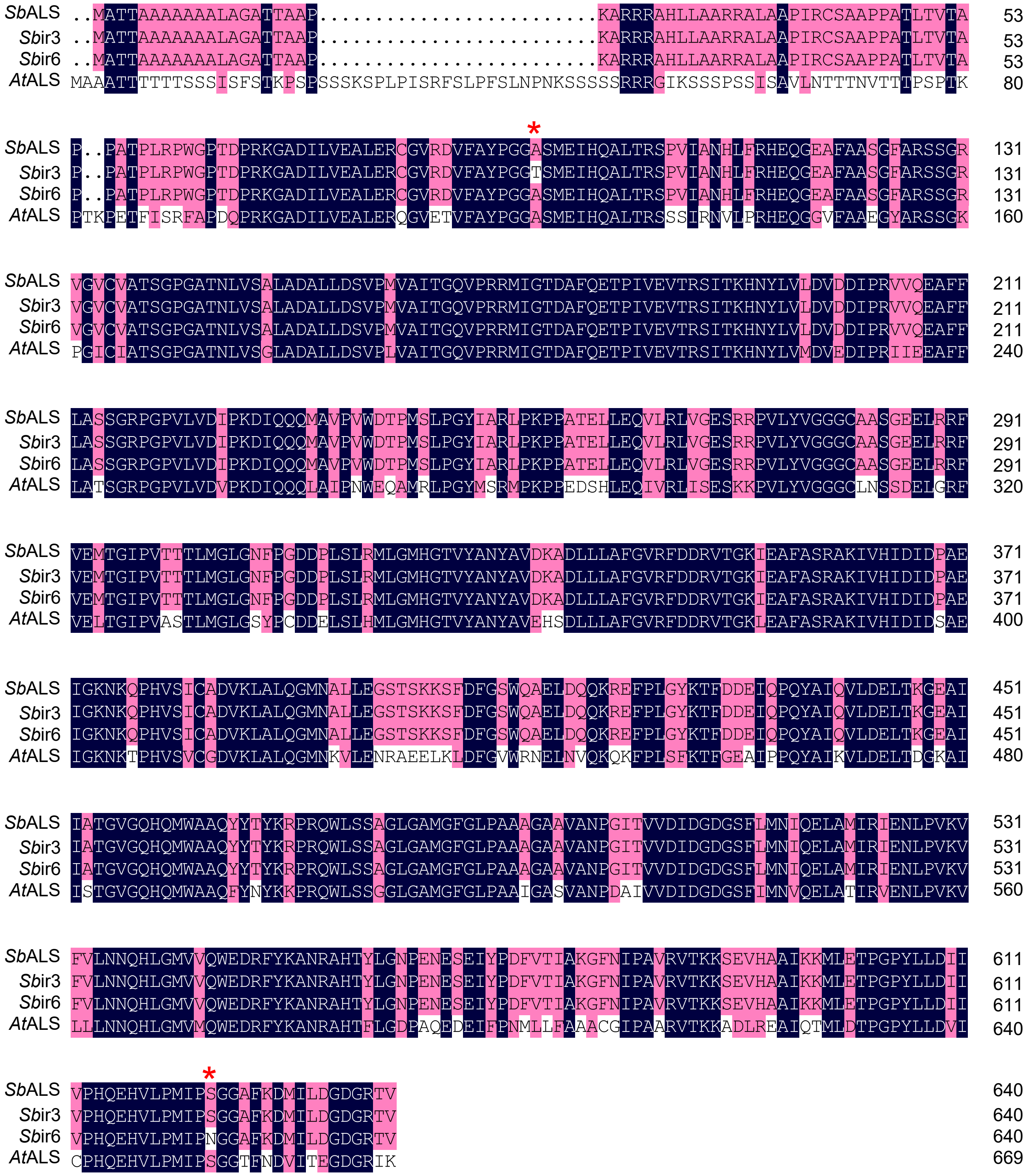

Figure S1 Protein sequence alignment of wide-type *Sb*ALS, *Sb*ir3, *Sb*ir6, and *At*ALS. The protein sequence identity between *Sb*ALS and *At*ALS was 66.17%. The asterisks indicate the mutated amino acid sites.

Figure S2

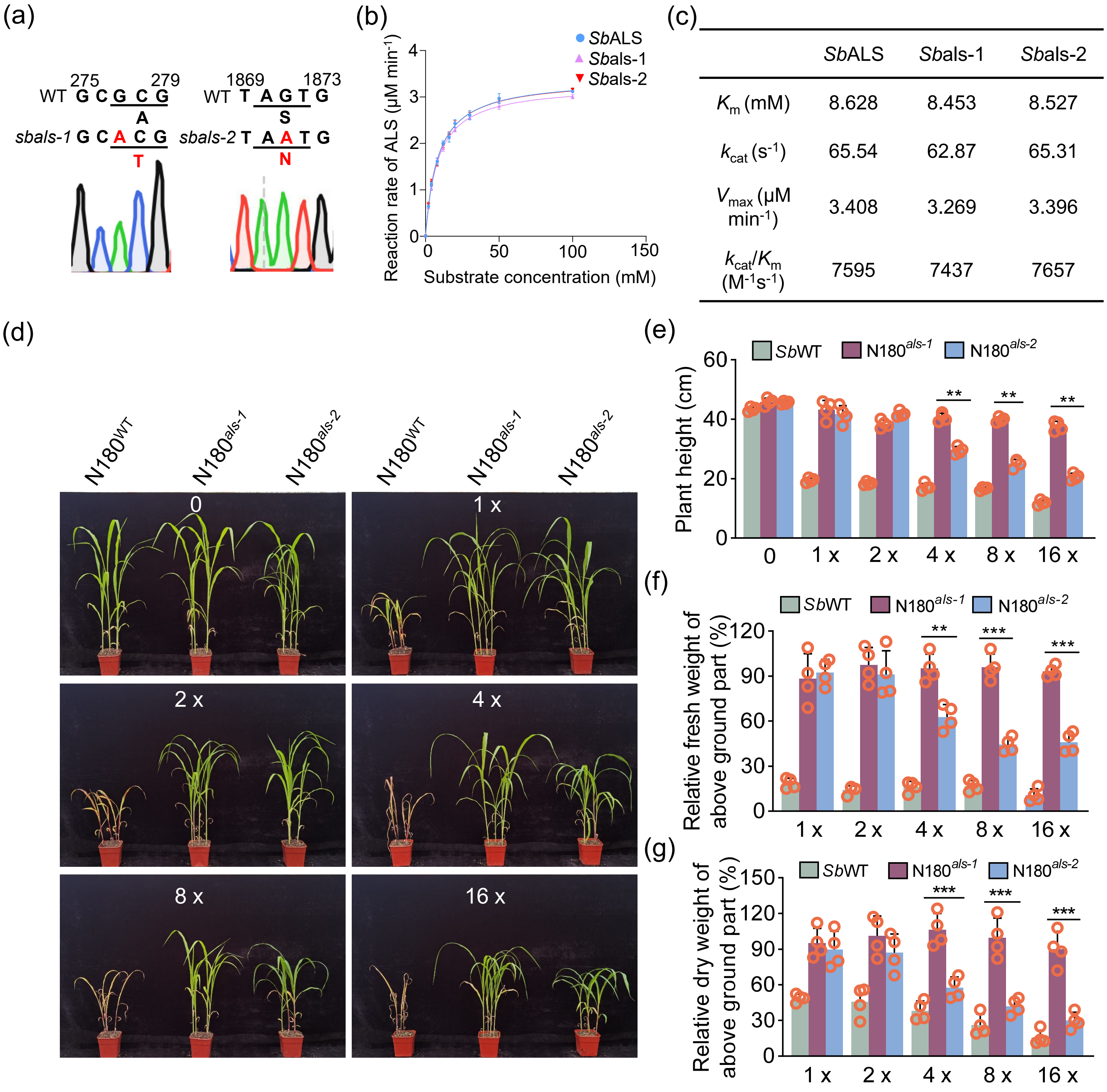

Figure S2 A93T confers imazamox-resistance in elite sorghum variety N180. (a) The G277A and G1871A mutations in *sbals-1* and *sbals-2* result in the amino acid substitutions A93T and S624N in the *Sb*ALS protein, respectively. *sbals-1* is homozygous for the G277A mutation in SbALS, and *sbals-2* is homozygous for the G1871A mutation in SbALS. The numbers indicate the positions of nucleotide bases in the SbALS open reading frames. (b) The enzyme activity assay of *Sb*ALS and mutants (*Sb*als-1 and *Sb*als-2). (c) Comparison of the kinetics based on mutant sites in *Sb*ALS. K_m is the Michaelis constant, V_{max} is the maximal reaction velocity and k_{cat} is the catalytic constant, where the k_{cat}/K_m value indicates the catalytic efficiency of the enzyme. (d) Phenotypic observation of N180/*sbals* BC_5F_3 plants treated with different concentrations of imazamox. (e-g) Analysis of the plant height, and relative fresh weight, and dry weight of aboveground parts of plants in (d). The mean fresh weights and dry weights (% of control) were calculated from three independent biological replicates. Error bars indicate the standard deviation (SD). n = 4. Asterisks in (e-f) denote statistical differences as determined by Student's *t*-test: $^{**}P < 0.01$ and $^{***}P < 0.001$.

Figure S3

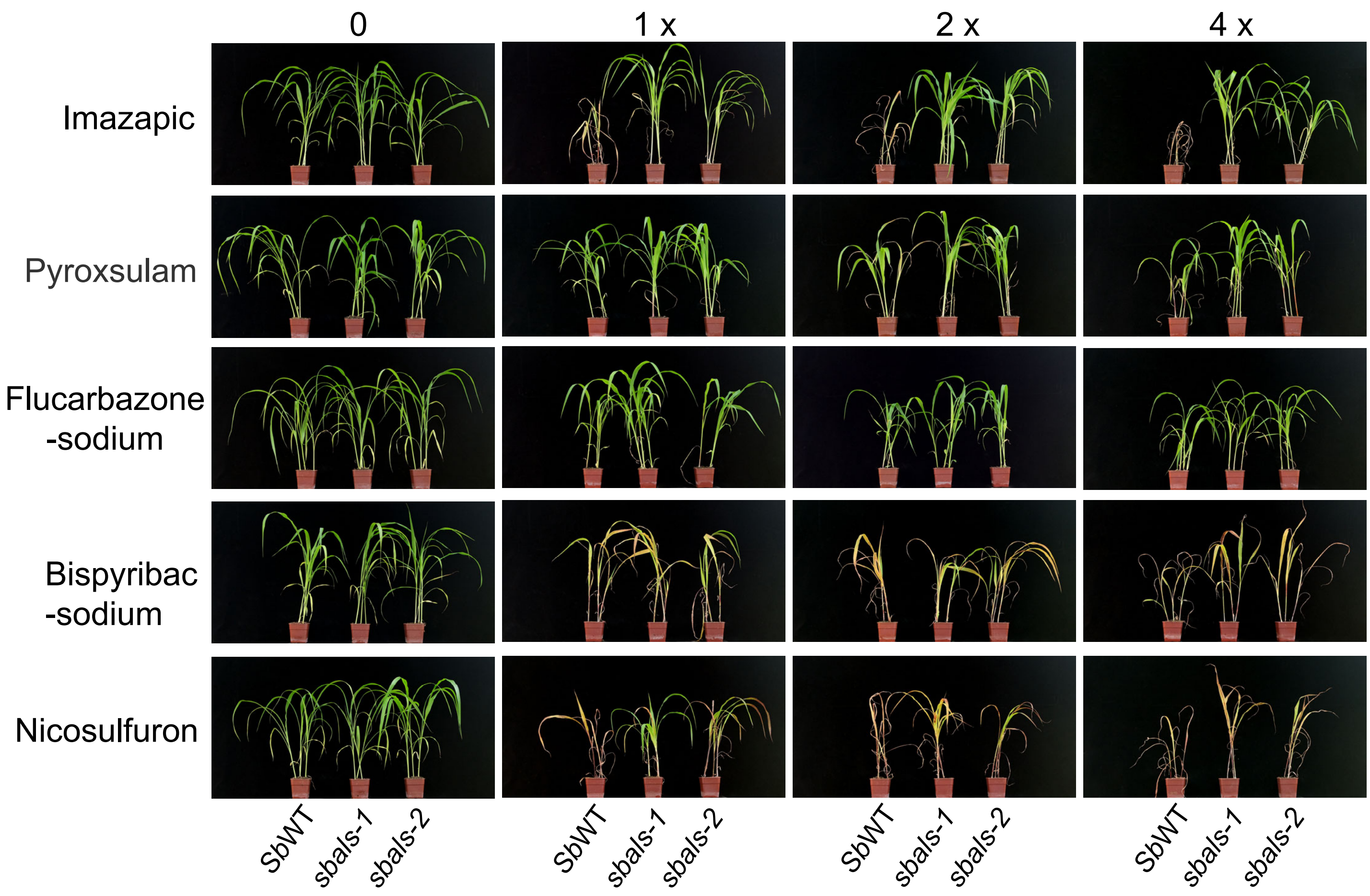

Figure S3 Comparison of resistance spectra and levels to various ALS-inhibiting herbicides among WT, *sbals-1*, and *sbals-2.* 1× represent the herbicide application dose equivalent to the recommended field-use. The recommended field doses for each herbicide are as follows: 108 g of active ingredient (a.i.) per hectare for imazapic, 9 g a.i./ha for pyroxsulam, 31.5 g a.i./ha for flucarbazone-sodium, 33.75 g a.i./ha for bispyribac-sodium and 54 g a.i./ha for nicosulfuron.

Figure S4

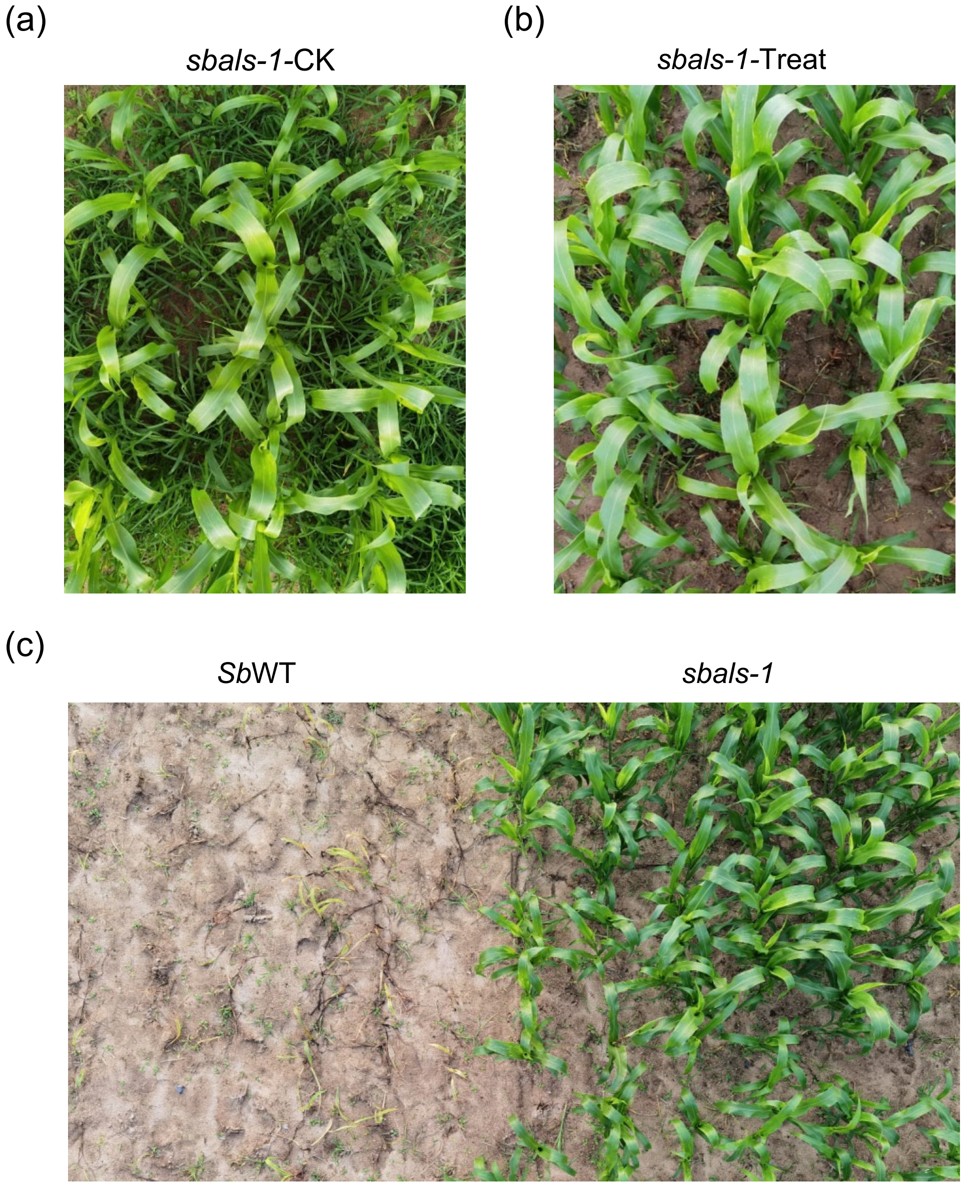

Figure S4 Herbicide response of *sbals-1* and *Sb*WT in Hainan. (a) Pre-treatment images of *sbals* plants before being treated with imazamox. (b) Post-treatment images of *sbals* plants after being treated with imazamox. (c) Comparison of SbWT and *sbals-1* plants treated with imazamox. All plants were photographed 10 days after spraying with 2× imazamox.

Figure S5

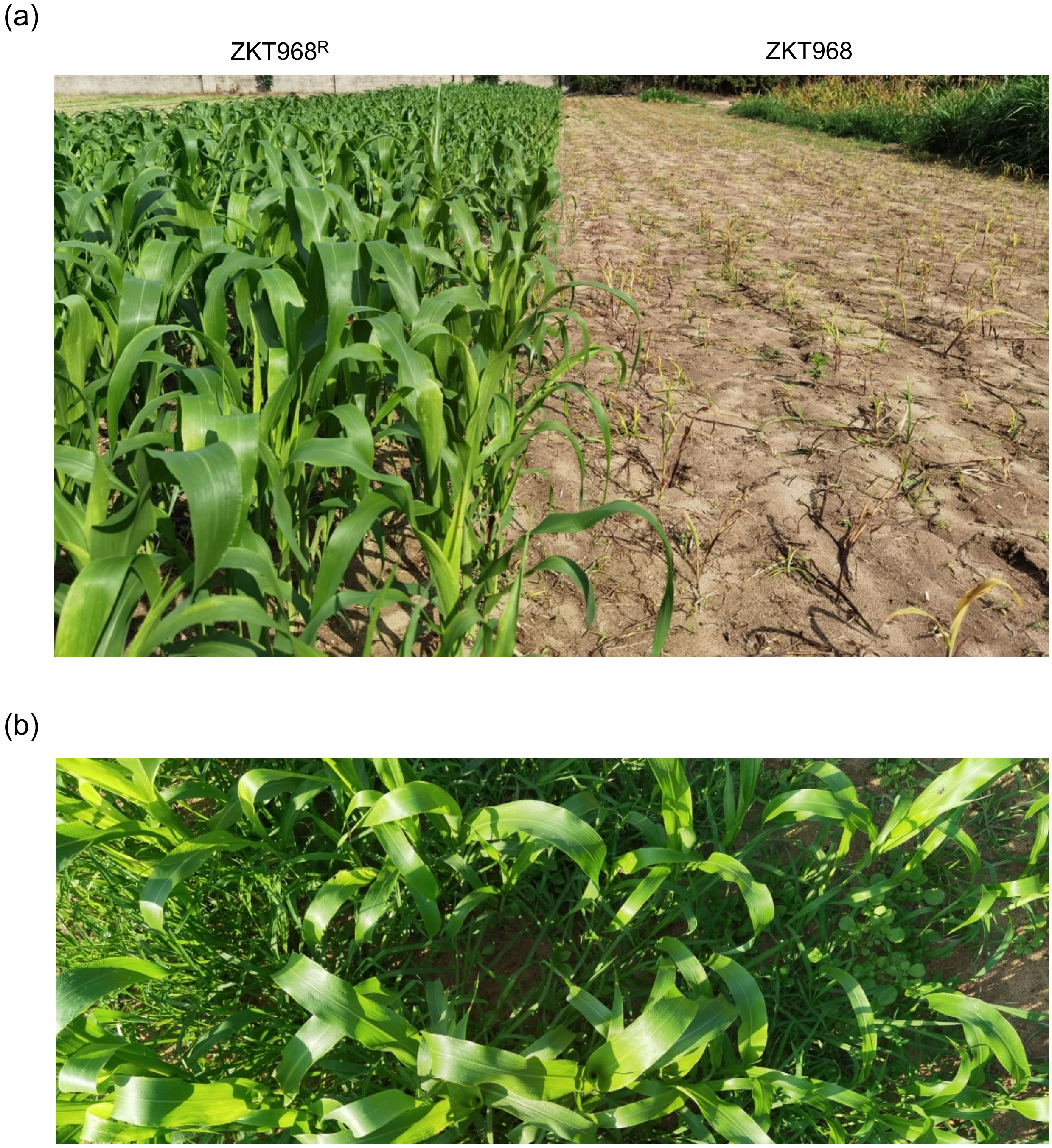

Figure S5 Herbicide response of ZKT968^{R} and ZKT968 in Hainan. (a) Homozygous ZKT968^{R} and ZKT968 plants treated with imazamox. All plants were photographed 10 days after spraying with 2× imazamox. (b) ZKT968^{R} plants control without imazamox treatment.

Figure S6

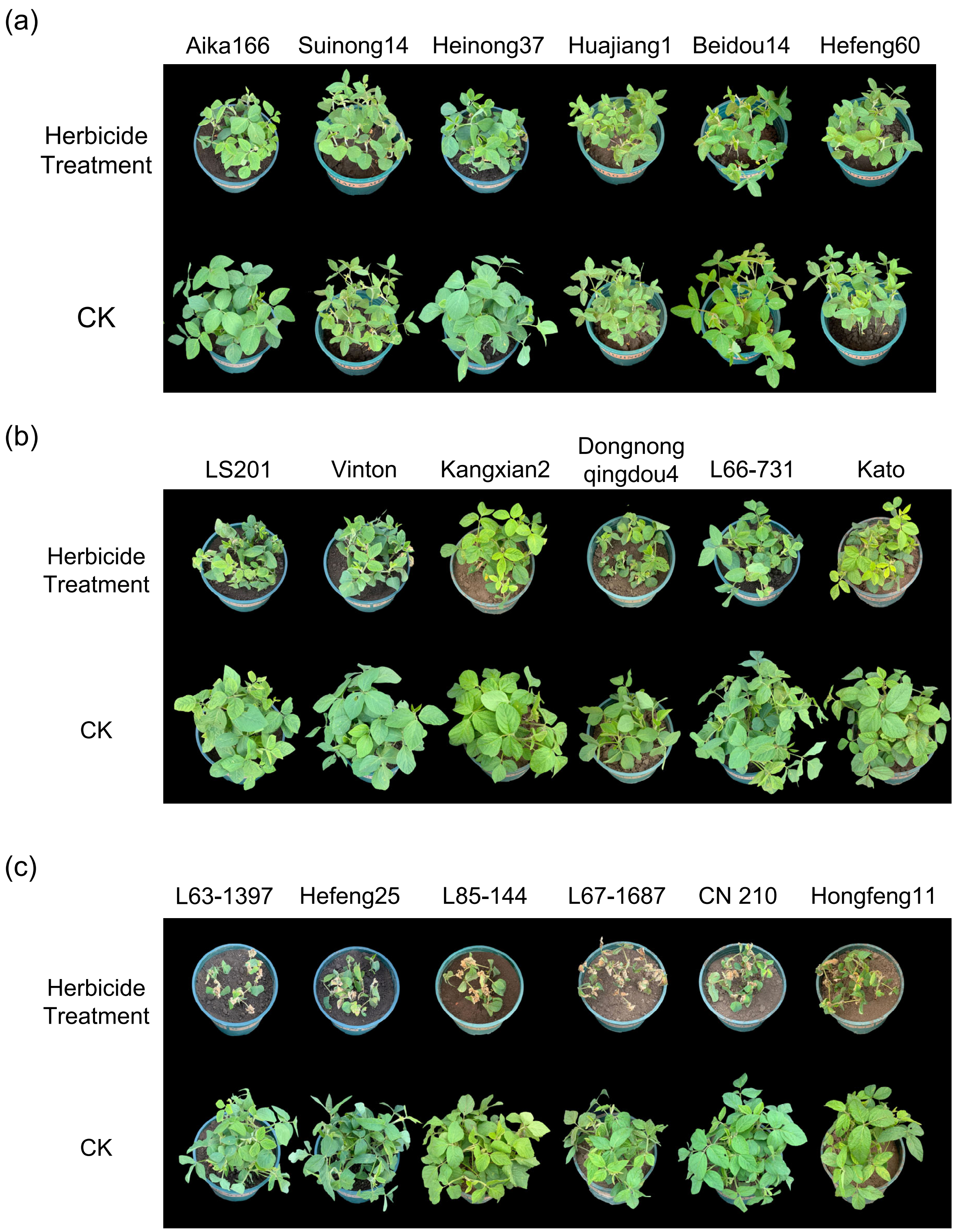

Figure S6 Representative soybean accessions with different levels of imazamox resistance. The level of imazamox resistance can be categorized as high tolerance (a), middle tolerance (b) and low tolerance (c) based on the herbicide resistance index.
